# Rapid phage susceptibility determination using a semi-automated bioluminescent ATP assay

**DOI:** 10.1128/spectrum.00782-26

**Published:** 2026-08-20

**Authors:** Houssein Chalhoub, Valéry Daubie, Hafid Dahma, Bob Blasdel, Isabelle Tytgat, Johan Quintens, Olivier Vandenberg, Marie Hallin

**Affiliations:** 1 Center for Environmental and Occupational Health, Ecole de Santé Publique - Université Libre de Bruxelles 81968, Brussels, Belgium; 2 Research & Innovation Unit, Laboratoire Hospitalier Universitaire de Bruxelles - Universitair Laboratorium Brussel (LHUB-ULB), Université Libre de Bruxelles 26659, Brussels, Belgium; 3 SYNLAB Belgium SRL, SYNLAB Laboratoire Collard, Liège, Belgium; 4 Department of Microbiology, Laboratoire Hospitalier Universitaire de Bruxelles - Universitair Laboratorium Brussel (LHUB-ULB), Université Libre de Bruxelles 26659, Brussels, Belgium; 5 Inteliphage, Mont-St-Guibert, Belgium; 6 Faculté de Médecine, Institute for Interdisciplinary Innovation in Healthcare (I3h), Solvay Brussels School of Economics and Management, Université Libre de Bruxelles (ULB) 37465, Brussels, Belgium; 7 European Plotkin Institute for Vaccinology - Université Libre de Bruxelles (EPIV-ULB) 26659, Brussels, Belgium; University of California San Diego, La Jolla, California, USA

**Keywords:** bacteriophages, phage susceptibility testing, clinical microbiology

## Abstract

Antimicrobial resistance is a major threat to human health. Phage therapy is a promising alternative to antibiotics, yet routine diagnostic tools capable of rapidly determining phage susceptibility are lacking. Our goal was to develop a diagnostic tool providing easy-to-interpret *in vitro* phage activity results within a clinically relevant time frame. We developed a prototype based on bioluminescence emitted by adenosine triphosphate (ATP) release during phage-mediated bacterial lysis. Ten phages were tested against 20 clinical isolates of four species: *Pseudomonas aeruginosa*, *Staphylococcus aureus*, *Escherichia coli*, and *Klebsiella pneumoniae*. Overall concordance with double agar overlay (DAO) was fair (72%). The prototype showed high sensitivity (94.5%) at detecting weak killers and high specificity (87%) at identifying strong killer phages. Most discordances involved DAO weak killers classified as active by the prototype. Most of these discordant phage-bacteria couples produced a spot in the spot test, suggesting the assay may detect but not distinguish killing without replication from killing with replication. The method showed high repeatability and reproducibility. A few aberrant species specificity results highlighted the need for routine controls. Results from prospective urine samples were obtained within an average of 5 h.

**IMPORTANCE:**

The lack of reproducible user-friendly phage susceptibility testing that can timely inform clinical decisions remains a major barrier to the routine clinical use of phages. We developed a high-throughput, semi-automated prototype that delivers easy-to-interpret phage susceptibility results for four bacterial species within 5 h in a “real-life” clinical flow. However, the correlation between *in vitro* lytic activity and clinical outcomes still needs to be established to meet regulatory requirements for *in vitro* diagnostic (IVD) medical devices.

## INTRODUCTION

As antimicrobial resistance (AMR) progresses worldwide, researchers are actively exploring alternatives to antibiotics. Among them, phage therapy is increasingly reported as an effective solution. However, for phage therapy to be considered a credible routine clinical alternative to antibiotics, it requires a robust *in vitro* phage susceptibility testing (PST) tool that complies with the requirements for *in vitro* diagnostic (IVD) medical devices. Developing a method that meets this need remains an unsolved challenge (1).

In contrast to antibiotics, which act as chemical agents that disrupt bacterial processes, bacteriophages replicate within bacterial hosts and use cell lysis to release their progeny, making PST far more complex than antibiotic susceptibility testing (AST). The life cycle of the obligately lytic tailed phages used in phage therapy involves several key steps, including virion interaction and adsorption to bacterial cells, nucleic acid translocation, a latent period, and finally progeny release (2). These steps, along with parameters such as phage burst size and relative phage-to-bacteria concentration (multiplicity of infection—MOI), play a critical role in determining the effectiveness of a given phage against a specific bacterial strain (3). Indeed, phages may kill bacteria without replicating, through mechanisms like abortive infection or lysis from without (4). Additionally, bacterial resistance mechanisms, such as receptor loss or CRISPR-based defenses, can interfere at various stages of infection. Since these factors can significantly impact clinical outcomes, it is important to use a testing method that can comprehensively index or at least estimate all these parameters (5).

Additionally, for practical and scalable routine clinical application, a PST should be accurate, reproducible, and robust, minimize the need for highly trained technical staff, allow random access, and provide unambiguous results that can be readily used in clinical decision-making. Finally, these results should be produced within a timeframe that is comparable to those of current AST methods.

There are two widely recognized reference methods for PST, the double agar overlay (DAO) and the spot test (ST), both based on the principle of challenging bacterial lawns with known phage concentrations. Both are time-consuming and require highly skilled operators. Both explore, in a 24- to 48-h time span, the phage-host interaction comprehensively, although the spot test indexes abortive infection or lysis from without, while DAO doesn’t (6), leading to potential discrepant results. From a clinical point of view, DAO indexes if a phage can be considered as an “active” treatment (when both lysis and *in situ* phage amplification occur), while ST indicates, as a proximate outcome, that the phage preparation can at least be used as a “passive” treatment, where only cell killing needs to occur (1). Finally, because both DAO plaques and ST spots can show a wide range of size, shape, and clearance, their interpretation is subject to interobserver variability, posing a challenge to their consistent and reliable use in routine clinical practice.

**Fig 1:**
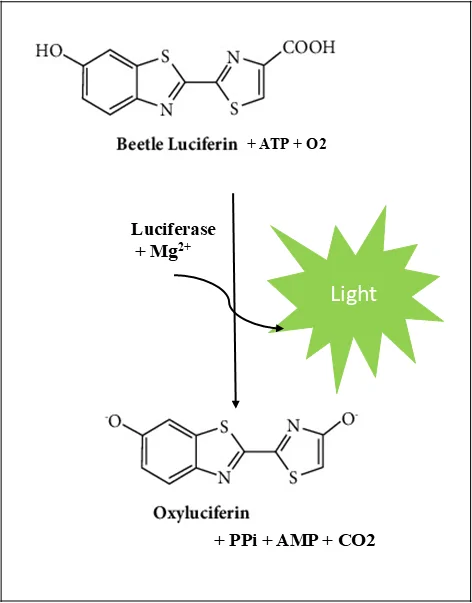
Prototype’s reaction principle. Firefly luciferase is a light-emitting enzyme responsible for the bioluminescence of fireflies. The oxidation reaction it catalyzes requires ATP, and the light emitted is directly proportional to the amount of extracellular ATP in the reaction medium, provided luciferin and luciferase are in excess. The bioluminescence has a broad visible spectrum, peaking at ~ 560 nm.

This work aimed to develop a user-friendly, high-throughput-compatible, and readily automatable assay capable of translating *in vitro* phage activity into actionable clinical decisions within a clinically relevant timeframe. We opted for a method based on bacterial lysis detection through extracellular adenosine triphosphate (ATP) quantification via luminescence measurement (Fig. 1). The resulting prototype provides easy-to-interpret phage susceptibility results within a 3-h hands-on time for four bacterial species designated by the WHO as priority AMR pathogens: *Pseudomonas aeruginosa* (*Pa*), *Staphylococcus aureus* (*Sa*), *Klebsiella pneumoniae* (*Kp*), and *Escherichia coli* (*Ec*). Additionally, we proved the method could be applied to bacterial pellets prepared from clinical urine samples, allowing the flexibility expected in clinical settings.

## MATERIALS AND METHODS

### Bacterial and phage strains

Phages and their reference host strains were provided by Inteliphage (at the time, Vésale Bioscience), Mont-St-Guibert, Belgium. The names of the phage-bacterial host pairs are listed in Table 1.

**TABLE 1:** Phage-bacterial host pairs used in this study

Phages	Bacterial hosts	
IPPA1	*P. aeruginosa*	*PAHS1*
IPPA3		
IPPA2		*PAHS2*
IPSA1	*S. aureus*	*SAHS1*
IPSA3		
IPSA2		*SAHS2*
IPKP1	*K. pneumoniae*	*KPHS1*
IPKP2		*KPHS2*
IPEC1	*E. coli*	*ECHS1*
IPEC2		*ECHS2*

A set of various bacterial and yeast reference strains (belonging to species most commonly found in clinical samples) were also used to test the species specificity of the prototype: *Streptococcus pyogenes ATCC19615*, *S. aureus ATCC29213*, *Staphylococcus epidermidis 49341308*, *Stenotrophomonas maltophilia ATCC13637*, *Serratia marcescens 49329145*, *Klebsiella oxytoca 2012B163*, *K. pneumoniae ATCC700603*, *Staphylococcus capitis ATCC27840*, *Staphylococcus haemolyticus ATCC29970*, *Candida krusei IHEM65610*, *Enterococcus faecium IOWA1*, *Haemophilus influenzae ATCC49766*, *Klebsiella aerogenes ATCC13048*, *P. aeruginosa ATCC27853*, *Proteus mirabilis 30650446*, *Enterococcus faecalis ATCC29212*, *Candida albicans ATCC24433*, *Staphylococcus lugdunensis ATCC43809*, *Streptococcus anginosus LMG14502*, and *Streptococcus agalactiae ATCC13813*. Additionally, the activity of each phage was evaluated and validated against a collection of 20 invasive clinical strains (blood culture isolates) of its bacterial host species.

### Reference methods

#### Double agar overlay

The titration of phage activity and calculation of the efficiency of plating (EOP) was performed as described in reference 7. Briefly, 100 µL of bacterial culture is mixed with 100 µL of serial 10-fold phage dilutions prepared in 0.9% NaCl, and 3 mL of soft (0.5%) agar in a test tube, followed by quick spreading onto a pre-warmed Tryptic Soy Agar plate. After allowing the agar to solidify for 10 min, the plates are incubated at 37°C for 18–20 h. Phage titer (plaque-forming unit [PFU]/mL) is then calculated based on the number of plaques observed on the plate by the dilution factor. EOP is defined as the titer of the phage on the bacterial cell studied relative to the titer observed with its reference strain (original bacterial host strain used for phage isolation and propagation during its initial characterization). An EOP classification, adapted from *in vivo* studies (8), was applied, with three predefined categories: phages with an EOP ≥0.1 were considered as “strong killers,” 0.005 < EOP < 0.1 indicated “intermediate killers,” and an EOP ≤0.005 corresponded to weak/no killing.

#### Spot test

ST was performed as described by reference 9. Briefly, bacterial cultures (100 µL) were added to 3 mL of pre-melted soft (0.5%) agar. The soft agar mixture was then poured onto a plain Luria-Bertani plate and allowed to solidify. A phage dilution series was prepared using 0.9% NaCl. Ten microliters of each selected serial 10-fold phage dilution was spotted onto the solidified soft agar and allowed to dry under the hood for 20 min. Plates were then incubated at 37°C overnight (18–20 h). The appropriate phage concentration for reading the plaque assay was determined by identifying the highest dilution that yielded countable plaques in the spot, relative to its reference strain. In cases where the DAO did not show any EOP or an EOP ≤0.005, the presence of a lytic spot was noted in order to index potential lysis from without (9). Additionally, a scoring of the morphology of the spot was performed as follows: clear lytic spot (LS); turbid LS: LS showing re-growing colonies; and faint LS: slight disturbance of the lawn (1).

### Prototype

#### Phage suspension preparation

For each phage, the working concentration was determined during a preliminary optimization phase by testing suspensions ranging from 10^6^ to 10^8^ PFU/mL against a panel of approximately 10–15 clinical isolates selected to represent a broad range of DAO results. The concentration yielding the most discriminative lysis scores (10^7^ or 10^8^ PFU/mL, depending on the phage) was retained for subsequent experiments.

#### Manual steps: bacterial pre-incubation and phage-bacteria co-incubation

A 0.5 McFarland suspension of the bacterial strain under investigation was prepared in a non-selective nutrient-rich liquid broth (LB) and incubated at 37°C for 120 min to allow bacterial activation in the culture medium.

**Fig 2:**
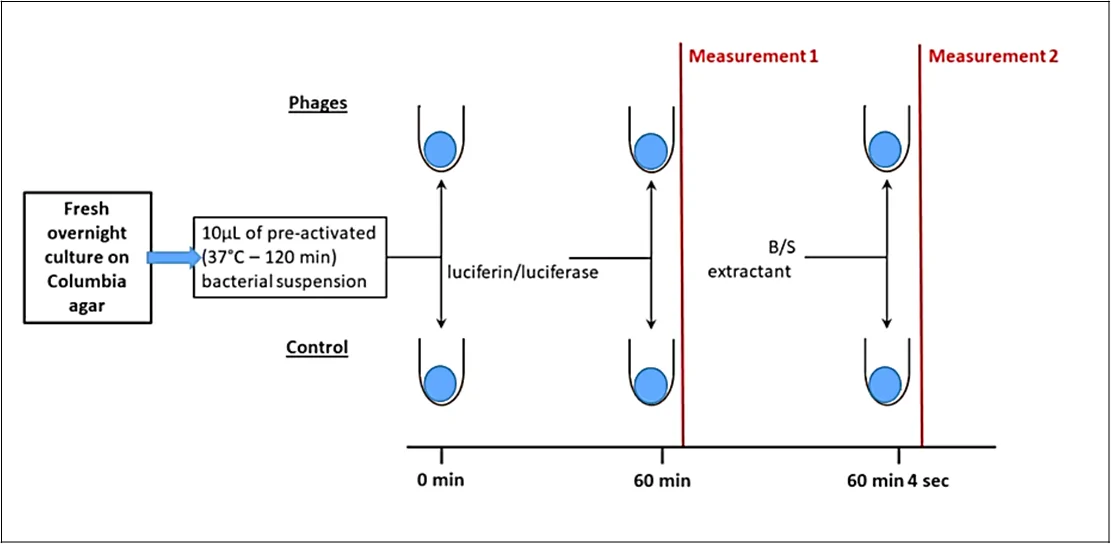
Schematic analytic process of the prototype. B/S extractant: lysis reagent.

After this “pre-incubation” step, this bacterial suspension was used to prepare two volume-to-volume mixtures: one with the phage suspension and the other, used as a control in the following steps, with sterile LB (Fig. 2).

These mixtures were then transferred into a 96-well, black, round-bottom microplate.

#### Automated light emission assay

Plates were incubated at 37°C for 60 min to allow phage-mediated bacterial lysis into a Centro LB 963 microplate luminometer (Berthold, Vilvoorde, Belgium). Light emission (visible emission spectrum, peaking at approximately 560 nm) was then measured twice in each well. The intensity of emitted light, measured in relative light units (RLU), was recorded to quantify the ATP released by lysed bacteria: the device first injected a luciferin/luciferase solution, followed by shaking and measurement of light emission. Magnesium required for luciferase activity was supplied by the luciferin/luciferase reagent, without additional magnesium supplementation. The first measurement aimed at measuring phage-induced lysis. The second light emission measurement was performed after the addition of chemical lysis buffer to perform a standardized chemical lysis of the living bacteria remaining in the well.

#### Concordance with reference methods

To assess the reliability of our prototype, we compared the results obtained with the prototype to those from the DAO reference method in a set of 20 clinical isolates of the 4 bacterial species studied, as well as with the different reference host strains (2 for each species) for a total of 220 phage/bacteria couples: 66 for *Pa* and *Sa* (3 phages each), 44 for *Ec* and *Kp* (2 phages each). Phage/bacteria couples exhibiting discrepant results were further explored using the ST reference method.

#### Prototype precision evaluation

For each phage examined, the precision performance of the prototype was determined:

##### Intra-run repeatability

Three bacterial strains (one of each DAO EOP category) were tested five times in a single run. The goal was to obtain a lysis score with a standard deviation (sd) below 10.

##### Inter-run reproducibility

The same three bacterial strains were tested across five runs carried out on five different days. The goal was to obtain a lysis score with an sd below 10.

##### Species specificity

Species specificity analyses were performed (in triplicate) for each phage against the reference strains collection described above.

### Statistical analysis

Statistical analyses were performed using Prism software, version 8.0.1.244 (GraphPad Inc., MA, USA). The data distribution was assessed using the Shapiro-Wilk test. Comparisons between two groups were conducted using either a *t*-test (for Gaussian distributions) or the Mann-Whitney test (for non-Gaussian distributions). Multiple group analyses were performed using ANOVA (for Gaussian distributions) or the Kruskal-Wallis test (for non-Gaussian distributions). Post hoc tests were performed using Tukey’s method after ANOVA, Dunnett’s test when comparing multiple groups to a control, and Dunn’s test after the Kruskal-Wallis test. When comparing multiple non-normal groups to a control, Dunn’s test with a Bonferroni or Holm correction was used.

To convert the prototype results into a categorical format allowing to distinguish strong and weak killers, the optimal thresholds were determined using IBM SPSS Statistics software by generating receiver operating characteristic (ROC) curves plotting the rate of true positives against the rate of false positives of the prototype output metric against the DAO categorical results described above (8).

### Adaptation of the prototype on bacterial pellets from clinical urine samples

#### Spiking negative urines

Negative urine samples were inoculated with different reference strains of *Pa*, *Sa*, *Kp*, and *Ec* to achieve a degree of positivity of ++ or +++ (approximately 0.5 to 1 × 10^8^ CFU/mL, respectively), using the SediMAX analyzer (Menarini Diagnostics). The bacterial pellet was immediately prepared by centrifuging 1.5 mL of the urine sample in Eppendorf tubes at 14,000 × *g* for 10 min (10).

Supernatants were discarded, and the pellets were used for preparing a 0.5 McFarland standardized suspension, used to inoculate 3 mL of LB for 120 min of bacterial pre-incubation at 37°C, which was then used for the prototype.

The prototype results obtained on pellets were compared with the prototype and the DAO results obtained for the same strains cultured on Columbia blood agar.

#### Prospective clinical positive samples

In the second step, a total of 20 positive (++ or +++) fresh midstream urine samples were prospectively collected from the routine workflow and, if positive with *Pa*, *Sa*, *Kp*, or *Ec* (reliable identification by MALDI-TOF on the pellet) (10), processed for PST (prototype and reference methods on pellets and on subcultures) analysis. The concordance between the prototype method and the reference methods was assessed, along with the “real-life” turnaround times for each approach.

## RESULTS

### Prototype development

#### Measurement of [ATP] after chemical and phage lysis

First, a standard curve linking RLU and [ATP] in LB was established to verify the linear relationship between them (Fig. S1). A correlation coefficient (R²) close to 1 was consistently reproduced across two independent trials (*n* = 4, each). Then, the spontaneous light emission in the LB medium alone, LB supplemented with phages, or intact, viable bacteria was studied over time (Fig. S2). Light emission was negligible for up to 120 min and not influenced by the presence of phages or bacteria. Furthermore, when fixed ATP quantities were added to these different mixes, measured [ATP] remained stable over time (Fig. S2).

We then measured [ATP] in LB containing viable bacteria as compared to the same amount of bacteria but chemically lysed; the sterile medium alone was used as a control (Fig. S3A). [ATP] in wells containing lysed bacteria was significantly higher than in wells containing viable bacteria or the medium alone. The same assay was then performed on reference strains in the presence or absence of their corresponding phages at 0 and 60 min (Fig. S3B). [ATP] in wells containing bacteria and phages was, at t0, similar to that observed in the absence of phages. After 60 min, [ATP] in wells containing phage-lysed bacteria was significantly higher than at t0 and in wells containing viable bacteria, in which [ATP] remained stable over time, showing the technique could index phage lysis, as well as chemical lysis.

Finally, [ATP] was measured over time and compared to bacterial colony count (CFU) for a bacterial strain incubated in the presence of a known strong killer phage and known weak killer phages against it (Fig. S4). [ATP] measured in the strong killer’s well significantly raised from 30 to 180 min of co-incubation, mirroring the CFU count falling during the same time frame, which was not the case in weak killer’s wells.

#### Bacterial pre-incubation

The effect of pre-incubating the bacteria in LB for 30, 60, and 90 min prior to phage addition was evaluated; the idea was to assess whether the growth phase of the bacteria at the time of initiating the phage-bacteria co-incubation could influence the performance of the method. The maximum incubation time tested was 2 h, as longer incubation time would potentially reduce its practicality for routine clinical applications. The results demonstrated that the amount of ATP released by phage lysis improved with a pre-incubation of up to 120 min (Fig. S5).

#### MOI determination

For each phage studied, an optimal bacteria/phage ratio was sought for the application of the method by varying the volumes of phages and bacteria added to the medium. A bacteria/phage ratio of 1 provides optimal values for *Pa* and *Sa* phages (Fig. S6). For *Ec* and *Kp* phages, an MOI of 0.1 was selected (data not shown).

#### Data standardization

Due to significant variability in RLU emissions across tests, batches of reagents and bacterial strains (11), we decided to include a control well (medium + bacteria, but no phage) in each run and, for “test” wells, to normalize the RLU released during phage-induced lysis against the RLU emitted in the same well by a standardized chemical lysis and express results as “lysis score” using the following formula:


$$\begin{array}{l} Lysis\;score \end{array}\;=\begin{array}{l} \frac{\left(RLU\;of\;the\;test\;well\;(bacteria\;with\;phage)\;after\;60\;min-RLU\;of\;control\;well\;(bacteria)\;after\;60\;min\right)}{\left(Re-measured\;RLU\;of\;the\;test\;well\;after\;addition\;of\;chemical\;lysis\;buffer-RLU\;of\;control\;well\;after\;60\;min\right)}\times 100 \end{array}.$$

This approach accounts for inter-test variability and provides a standardized measure of phage-induced lysis. In the subsequent analysis performed, the value of the score usually ranged between 1 and 100, although exceptional results below 0 were observed for weak/no killers, and scores above 100 occasionally occur for very strong killers.

### Concordance with reference methods

#### Prototype vs DAO categories

**Fig 3:**
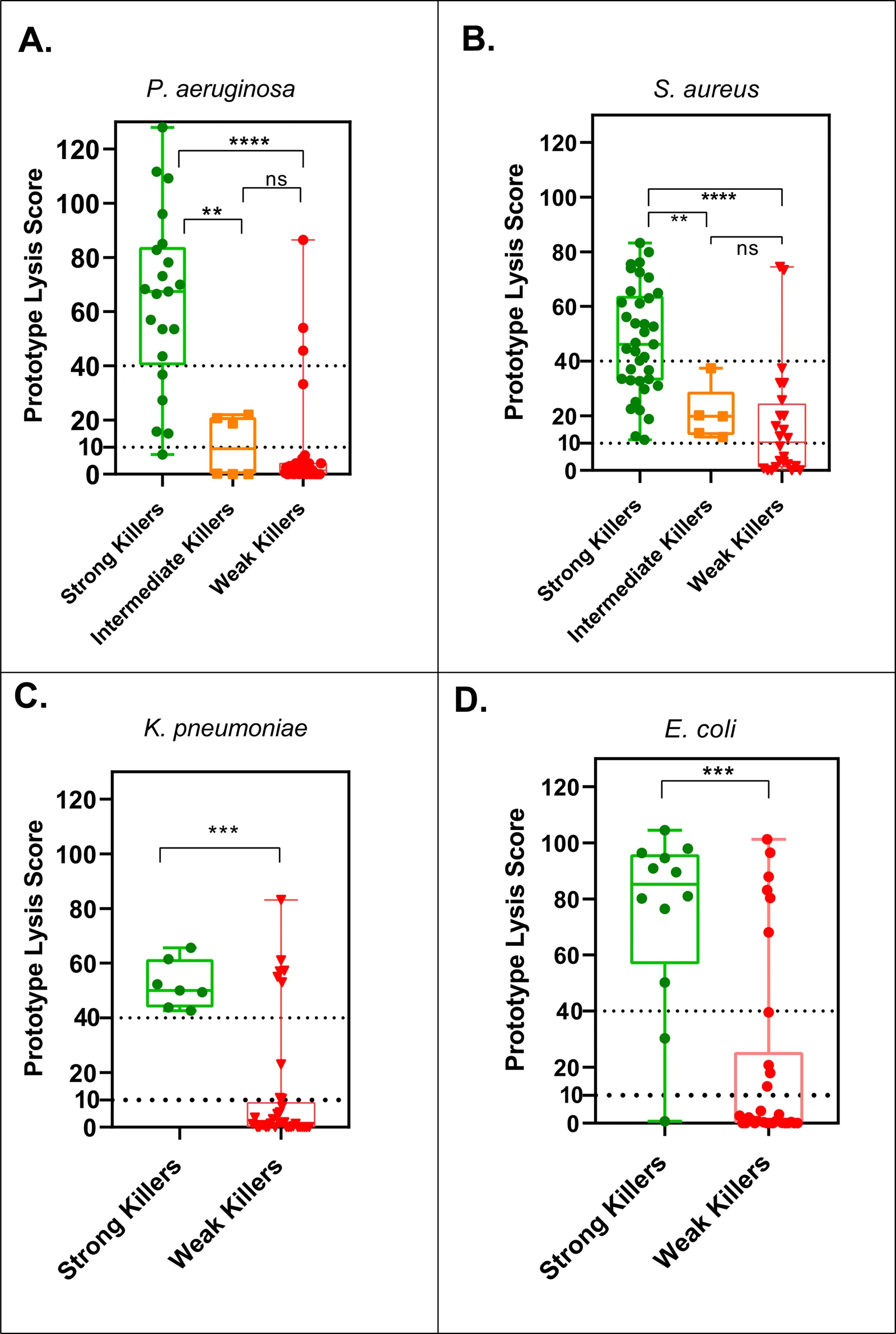
Lysis score obtained with the prototype as compared to classification of the EOP obtained by DAO in categories (8): EOP ≥ 0.1 for strong killers, 0.005 < EOP < 0.1 for intermediate killers, EOP ≤ 0.005 for weak/no killers) for (**A**) *P. aeruginosa* (3 phages, 66 bacterial strain-phage pairs), (**B**) *S. aureus* (3 phages, 66 bacterial strain-phage pairs), (**C**) *K. pneumoniae* (2 phages, 44 bacterial strain-phage pairs), and (**D**) *E. coli* (2 phages, 42 bacterial strain-phage pairs, 2 intermediate killers are not represented). Boxes indicate median, 25th and 75th percentiles; whiskers indicate minimum and maximum values; **: *P* value < 0.01; ***: *P* < 0.001; ****: *P* < 0.0001; ns, non-significant. See Table S1 for raw data and detailed statistics.

We compared the lysis score obtained with the prototype to the EOP obtained by DAO for a set of 220 phage/bacteria couples across the four bacterial species studied (Fig. 3; Fig. S7). Lysis scores observed for phages classified as strong killers by DAO were significantly higher than scores displayed by DAO weak/no killers. Strong killer scores were also significantly different from those of the intermediate killers’ classes in *Pa* and *Sa* (Fig. 3). This couldn’t be studied for *E. coli* and *K. pneumoniae* phages, as few intermediate killer results were observed for these two species (*Ec*, *n* = 2; *Kp*, *n* = 0).

**Fig 4:**
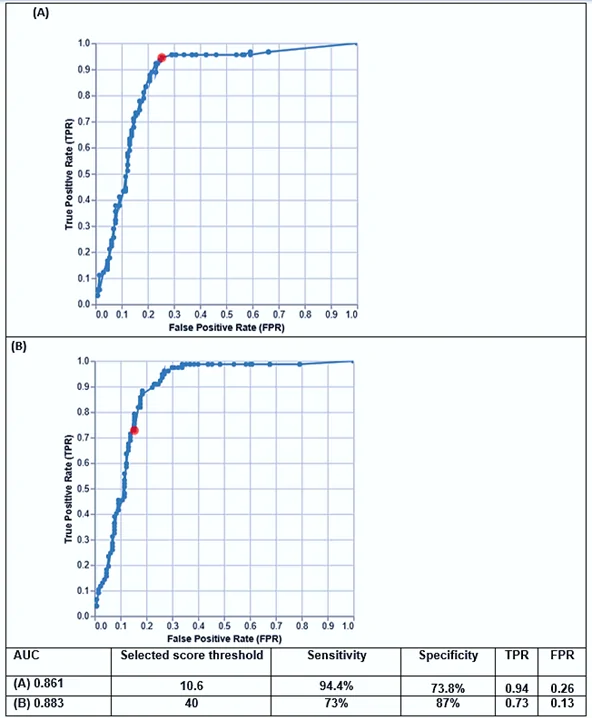
ROC curves of lysis score against DAO categories. (**A**) Weak killers (yes/no), and (**B**) strong killers (yes/no), in all species (Gram-negative and positive strains). The threshold selected is highlighted in red.

To ease the translation of the lysis score into standardized clinical actions, we decided to classify scores in a categorical format comparable to the “sensitive, intermediate, resistant” (SIR) classification currently used to report antibiotic activity against bacteria. To this end, we established two ROC curves plotting the prototype lysis scores against DAO categorical results (Fig. 4). As expected, the area under the curve values were both above 0.85, fitting for very good performance. We selected the score of 10 as the threshold for excluding weak- to non-lysing phages. The score of 40 was selected as the threshold categorizing strong lysis activity. Our goal was to identify weak killers with a high sensitivity (94%) and acceptable specificity (74%), and strong killers with a high specificity (87%) and acceptable sensitivity (73%). Using these thresholds over the 220 couples studied, the global concordance of the prototype with DAO was 72% (159/220): 73% (56/77) in identifying strong killers, 61% (8/13) in identifying intermediate killers, and 73% (95/130) in identifying weak killers.

#### Prototype vs ST results in low EOP cases

**Fig 5:**
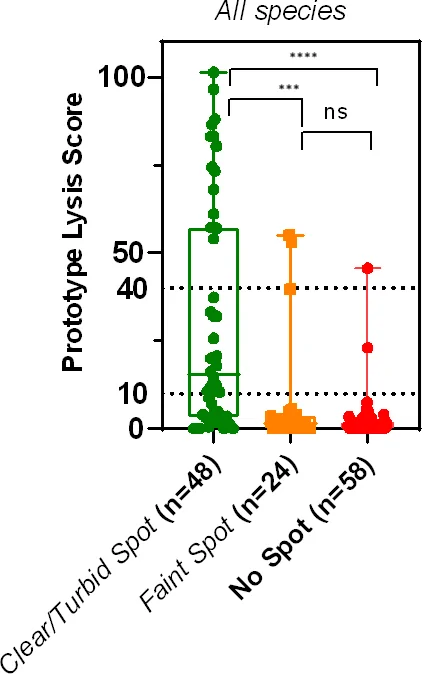
Lysis score obtained with the prototype as compared to ST results for weak killers as categorized by Green, Kaelber et al. (8) based on DAO (EOP ≤ 0.005) for all species (10 phages, 130 bacterial strain-phage pairs). Boxes indicate median, 25th and 75th percentiles; whiskers indicate minimum and maximum values; ***: *P* = 0.0001; ****: *P* < 0.0001; and ns, non-significant.

As illustrated in Fig. 3, bacteria-phage couples showing strong killing activity by DAO but a lysis score below 10 were exceptional (*n* = 2/220). By contrast, numerous couples showing an EOP <0.005 by DAO did show lysis scores above 10 (*n* = 35): 18 from 10 to 40 (moderate lysis), 17 above 40 (strong lysis). To explore these discrepancies, we explored these couples (Fig. 5; Fig. S7) using the ST reference method, and the vast majority (86%, 30/35) did exhibit activity through either a clear or a turbid spot.

### Analytical validation of the prototype

#### Repeatability and reproducibility

The observed lysis scores showed an sd that remained below the threshold of 10 around the mean at weak, intermediate, and strong activity values for all *Pa* (*n* = 3), *Sa* (*n* = 3), *and Kp* (*n* = 2) phages tested. Regarding *Ec* phages, one out of the two phages tested (IPEC2) showed, at the chosen working concentrations (10^7^ pfu/mL), an sd of 20 for strong and 11 for intermediate activity values.

#### Species specificity

Species specificity was performed for each phage against a reference bacterial and yeast strains collection to ensure that the prototype didn’t give any lysis score result above 10 in case of incubation of a phage with a bacterial species this phage is not directed against. An example is illustrated in Table S1 for IPSA3, active against *S. aureus* and other *Staphylococci*. This investigation identified several outliers (aberrant results), mainly in *H. influenzae* (IPSA3 score 73), Streptococci (*S. anginosus*, −222), and Candida species (*C. krusei*, 18). RLU analysis indicated that these were due to insufficient chemical lysis (Table S1), highlighting the need to verify both adequate growth and effective control-well lysis before interpreting test results.

### Implementation of the prototype on urine samples bacterial pellets

We first spiked fresh negative urine samples with bacterial isolates of each bacterial species studied and tested the pellet obtained using known active phages. The results were compared to those obtained with both the prototype and the DAO from pure culture of the same isolate on blood agar. The prototype and DAO consistently agreed on the activity category of the phages tested, regardless of the substrate used (solid culture or pellet) (Table S2).

We then prospectively collected 20 positive urine samples. Ten of them were reliably identified as *Kp* (*n* = 1) or *Ec* (*n* = 9) by MALDI-TOF on the pellet and consequently processed for PST (one phage/sample). Three samples were positive for a bacterial species that was outside the study scope; other pellets did not yield reliable identification. Prototype results classification was concordant between pellet and subculture and in agreement with DAO in all cases. Results of the prototype on pellets were available within an average of 5 h after the urine samples bacterial count. This was 16 h earlier than the DAO performed on the pellet and 38 h before routine AST results, performed on subculture (Table 2).

**TABLE 2:** TAT and PST results of prospectively collected positive urine samples

No. of samples	Mean TAT (h) (min/max)							Categorical concordance^*a*^ (%)		
	MALDI-TOF		Prototype		DAO		AB	Prototype vs DAO (pellet)	Prototype (pellet) vs DAO (subculture)	Prototype (pellet vs subculture)
	Pellet	Subculture	Pellet	Subculture	Pellet	Subculture	Subculture			
10	1.5	23.5 (20.5/28.5)	5.2	27.2 (25.2/29.2)	21.2 (20.2/22.2)	45.2 (44.2/46.2)	43.2 (35.2/49.2)	100	100	100

^*a*^ 1 *K. pneumoniae* strain (IPKP1 phage, weak killing), 9 *E. coli* strains (IPEC1 phage, 3 strong killing, 6 weak killing).

## DISCUSSION

Among the multiple strategies explored to tackle AMR, phages are promising: they present an effective bactericidal effect with a very narrow spectrum and very few side effects (12). This narrow spectrum, considered as an advantage over antibiotics regarding both microbiota preservation and emergence of resistance (13), comes with a couple of obstacles to their routine clinical use, such as the current lack of PST techniques able to meet the requirements for IVD medical devices (1).

We developed a semi-automated diagnostic prototype capable of multiplex determination of bacterial sensitivity to various phages within a 3-h hands-on time. Using affordable materials and instruments, this prototypic assay, performed on 96-well microplates, allows for both high throughput and random access. Additionally, a comparable bioluminescence-based technique has been previously successfully tested on uropathogens for AST (14), allowing possible future development for phage-antibiotic association testing. We chose to express results as a standardized “lysis score” that can be further classified in a categorical format comparable to the SIR classification currently used for AST. The prototype, tested using 10 phages against four bacterial species from the WHO’s list of priority AMR organisms, demonstrated global concordance with the DAO reference method and demonstrated both a high sensitivity (94.5%) at detecting weak killers and a high specificity (87%) at identifying strong killer phages. Most discrepancies between the prototype and DAO were observed with DAO weak killers presenting an “active” lysis score with the prototype. The vast majority of these discordant phage-bacteria couples also produced a spot with the ST method, suggesting that the prototype assay, similarly to ST, is likely unable to distinguish between bactericidal activity without phage replication and activity involving both bacterial killing and phage replication (4). Similar results were recently obtained by Bayat et al., who developed an all-in-one solid tablet platform utilizing ATP bioluminescence for rapid phage screening, making the authors claim their method to be as efficient as the ST assay, while offering the advantage of being rapid and high throughput (15). While their technology focuses on stabilizing decentralized phage biobanks through sugar-matrix encapsulation, our liquid-based prototype focuses on clinical integration into routine hospital workflows by delivering standardized SIR-like lysis scores from clinical samples within a clinically relevant timeframe. Together, these methodologies demonstrate the versatility of ATP-bioluminescence; where tablet-based arrays optimize biobank distribution and screening, our microplate prototype provides a practical, low-cost framework for rapid, real-time diagnostic decision-making at the point of care.

We further performed a thorough analytical performance assessment of the prototype. Our method was highly repeatable and reproducible, except for high lysis activity values of one *Ec* phage. The species specificity investigation, on the other hand, led to a couple of aberrant results. Species specificity testing is rarely performed when evaluating new PST methods but is of high importance regarding any clinical microbiology diagnostic test. It explores potential cross-reactions or false positive results that might occur while accidentally using mixed cultures, which is not a rare event in a routine setting. These findings, alongside the known inter-instrument and inter-run variability of “crude” RLU emission values due to environmental factors (such as temperature [16], pH, or presence of inhibitors [17]), highlight the need to ensure that effective controls are performed in each run, another key element for any IVD method applied to clinical use. Furthermore, both inter-instrument and inter-laboratory ring tests should be conducted (18). More generally, and as new PST techniques are constantly emerging (1), establishing a set of critical analytical parameters for their evaluation becomes a pressing need (18–20). Although several initiatives have been undertaken, no consensual reference standard is currently available. This lack of standardization hinders the objective evaluation and comparison of methodologies across studies. Indeed, numerous studies have, like ours, highlighted gaps in agreement of PST assays and approaches (18). While further studies on phage biology may help identify which PST methods most accurately capture the complexity of phage-bacteria interactions, the greatest unknown remains their predictive value to determine a phage’s *in vivo* efficacy (15, 19). Indeed, interpretive criteria or breakpoints for PST would ideally, like for AST, be determined based on clinical outcome (19); this is even mandatory to comply with EU Regulation on IVD medical devices (EU-IVDR) (1).

Historically, to achieve a timely administration of treatment, clinical trials have mainly evaluated empirical administration of fixed, spectrum-optimized cocktails of phages, the *in vitro* evaluation of their activity against each patients’ bacterial strain being performed retrospectively. This approach has led several cohort studies to fail to prove the effectiveness of phage therapy for the application studied because the cocktails too often had insufficient activity against the patients’ strain (1, 20). We sought to evaluate the integration of our prototype into a routine clinical workflow to assess how its 3-h hands-on time would translate into real-life time-to-results using urine samples. Urinary tract infections (UTIs) are one of the most prevalent bacterial infections globally (estimated 400 million cases/year), and are prone to become both recurrent and antibiotic resistant (13). A recent Italian prospective multicenter study reported a 39% prevalence of MDR pathogens in patients admitted to the emergency department for UTI, mainly *Ec* (57%) and *Klebsiella* spp. (24%) (21). In this regard, phages could rapidly become either an alternative or a complement to antibiotics (13). The results of our prototype were available on an average of 5 h after the bacterial count and were 100% concordant with the DAO results released 16 h later. These results, although derived from a small cohort, provide preliminary proof-of-concept evidence that an IVDR device based on this prototype may achieve robust PST results within a clinically realistic timeframe. While total automation and standardization of phage inocula in an “easy-to-store, ready-to-screen” microtiter plate proposed by others (15, 22) would further improve both the hands-on time and manual error-prone steps of the method, such devices could be of interest as a pre-administration screening in the frame of clinical trials evaluating phage therapy in UTIs.

### Conclusion

The lack of rapid, easy-to-use, and reproducible PST producing results that can be timely translated into clinical decisions is currently hampering phages from becoming a credible “routine” clinical alternative to antibiotics. It also impairs clinical studies evaluating phage therapy for human use. We describe here an affordable bioluminescence-based prototypic technique allowing for both high-throughput and random access that might help to fill this gap. Results, available in categorical format, showed to be highly sensitive at detecting weak killers and highly specific at identifying strong killer phages for four bacterial species (*Pa*, *Sa*, *Ec*, and *Kp*) within 5 h in a “real-life” clinical flow. Further improvements, such as full automation or standardization of phage inocula, are still needed for this prototype to comply with the EU-IVDR; but the main unknown that remains is—as for any PST—the correlation between the *in vitro* lytic activity it indexes and actual clinical outcome.

## Data Availability

The data sets generated and/or analyzed during the current study are not publicly available due to intellectual property protection, as this work led to an international patent issued on 2022-08-11 to Vesale Bioscience: WO2022167597 (A1); Automatic phagogram; Lienart van Lidh de Jeude J., Quintens J., Blasdel B. Retrieved on 6 December 2025, from https://be.espacenet.com/publicationDetails/biblioDB=EPODOC&II=0&ND=4&adjacent=true&locale=fr_BE&FT=D&date=20241127&CC=WO&NR=2022167597A1&KC=A1#. Requests for access will be considered only where compatible with intellectual property rights and applicable legal or contractual obligations.

## SUPPLEMENTAL MATERIAL

Supplemental tables and figures spectrum.00782-26-s0001.pdf Fig. S1 to S7, and Tables S1 and S2.

ASM does not own the copyrights to Supplemental Material that may be linked to, or accessed through, an article. The authors have granted ASM a non-exclusive, world-wide license to publish the Supplemental Material files. Please contact the corresponding author directly for reuse.
