# Supplementary material for: Rapid phage susceptibility determination using a semi-automated bioluminescent ATP assay
Source: Microbiol Spectr. 2026 Aug 20;14(10):e00782-26. doi: 10.1128/spectrum.00782-26 (PMC13689462; doi:10.1128/spectrum.00782-26)
Supplement: Supplementary file 1 [file spectrum.00782-26-s0001.pdf]

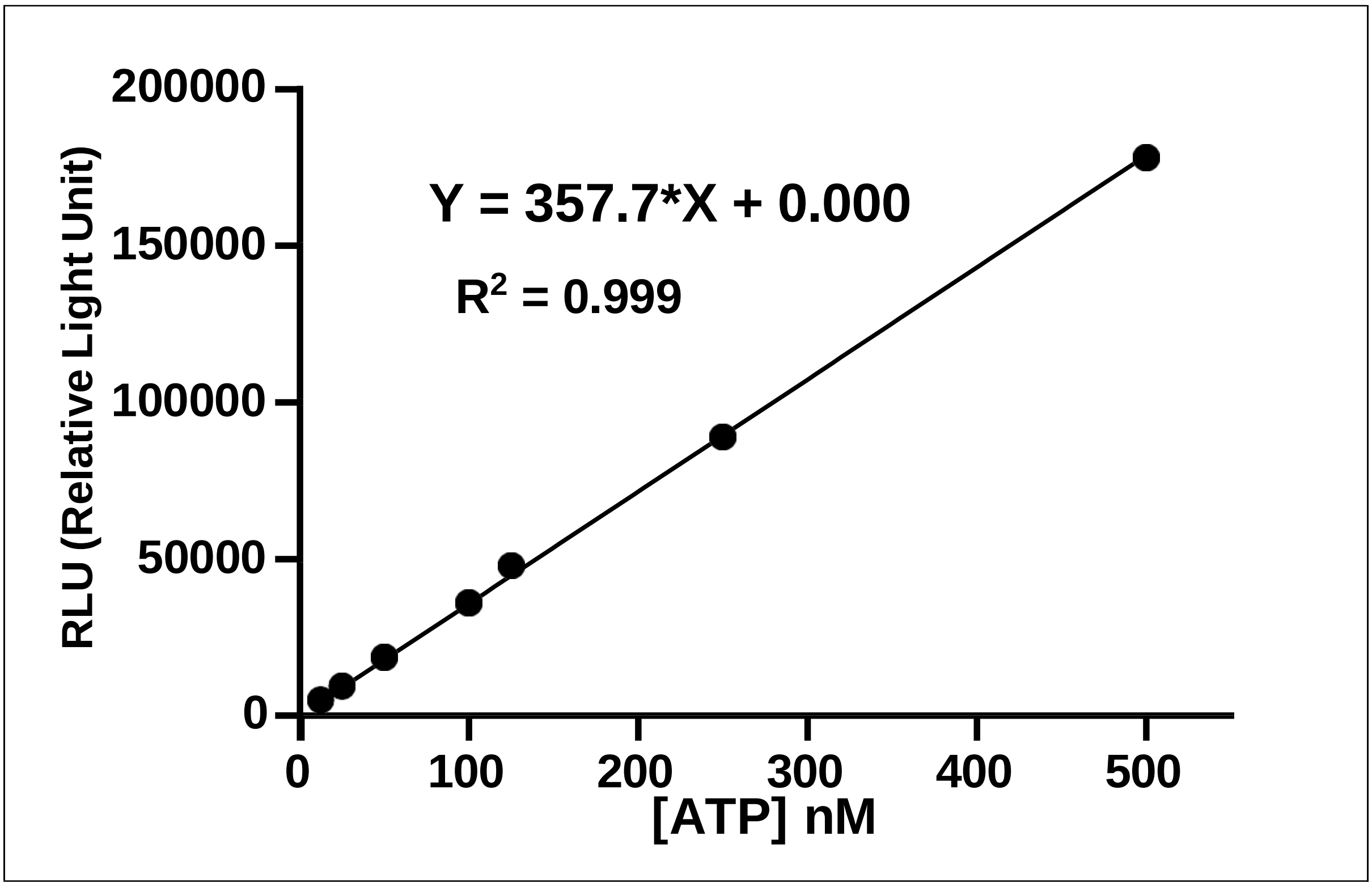

Figure S1: Light emission measured (RLU, Relative Light Unit) in function of ATP concentrations in liquid broth, performed in quadruplicates; results are expressed as means with standard deviations (SD) (SD bars included, but not visible as SD were negligible).

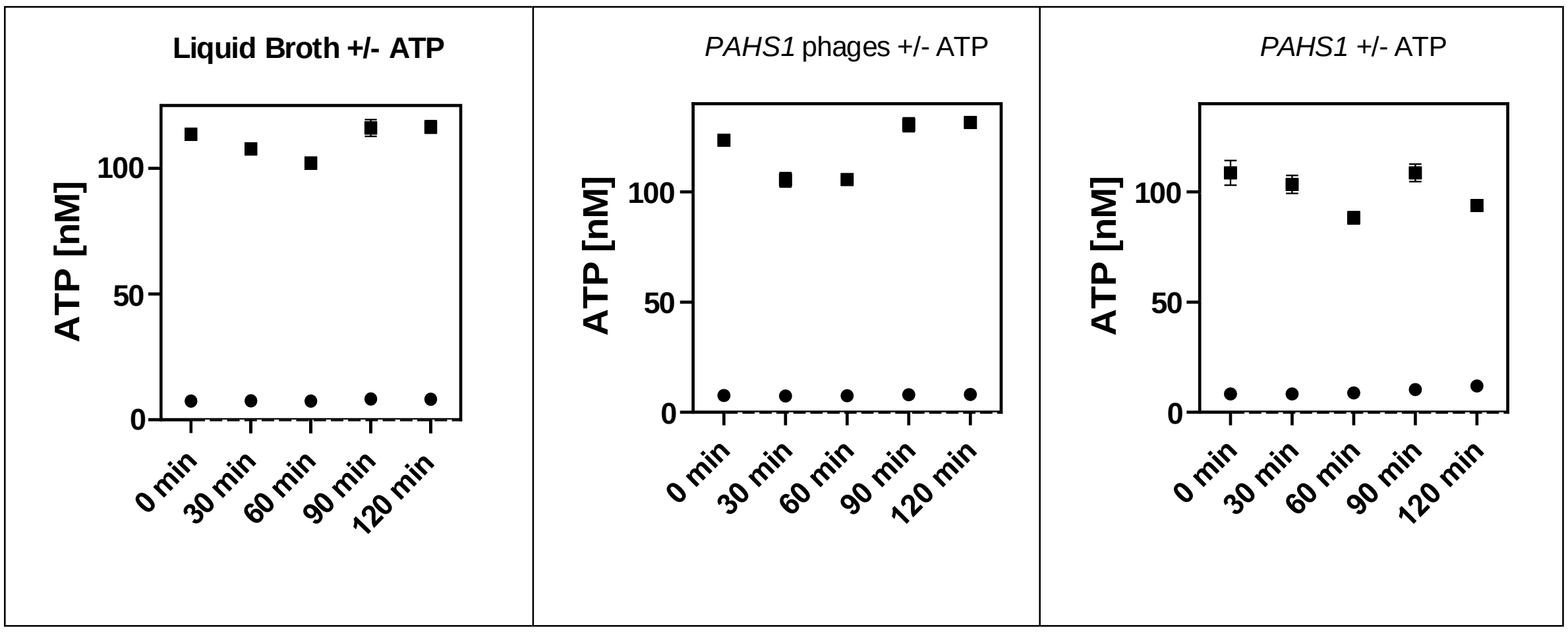

Figure S2: Stability of light spontaneously emitted by (Left) Liquid Broth (LB) alone, (Middle) LB supplemented with 10^8 PFU/mL of phage IPPA1, (Right) LB with 10^8 CFU/ml of the phage reference bacterial host strain, with (squares) and without (circles) the addition of [~100 nM] of ATP. Each was performed in triplicate, results are expressed as means with standard deviations (SD) (SD bars included, but not always visible).

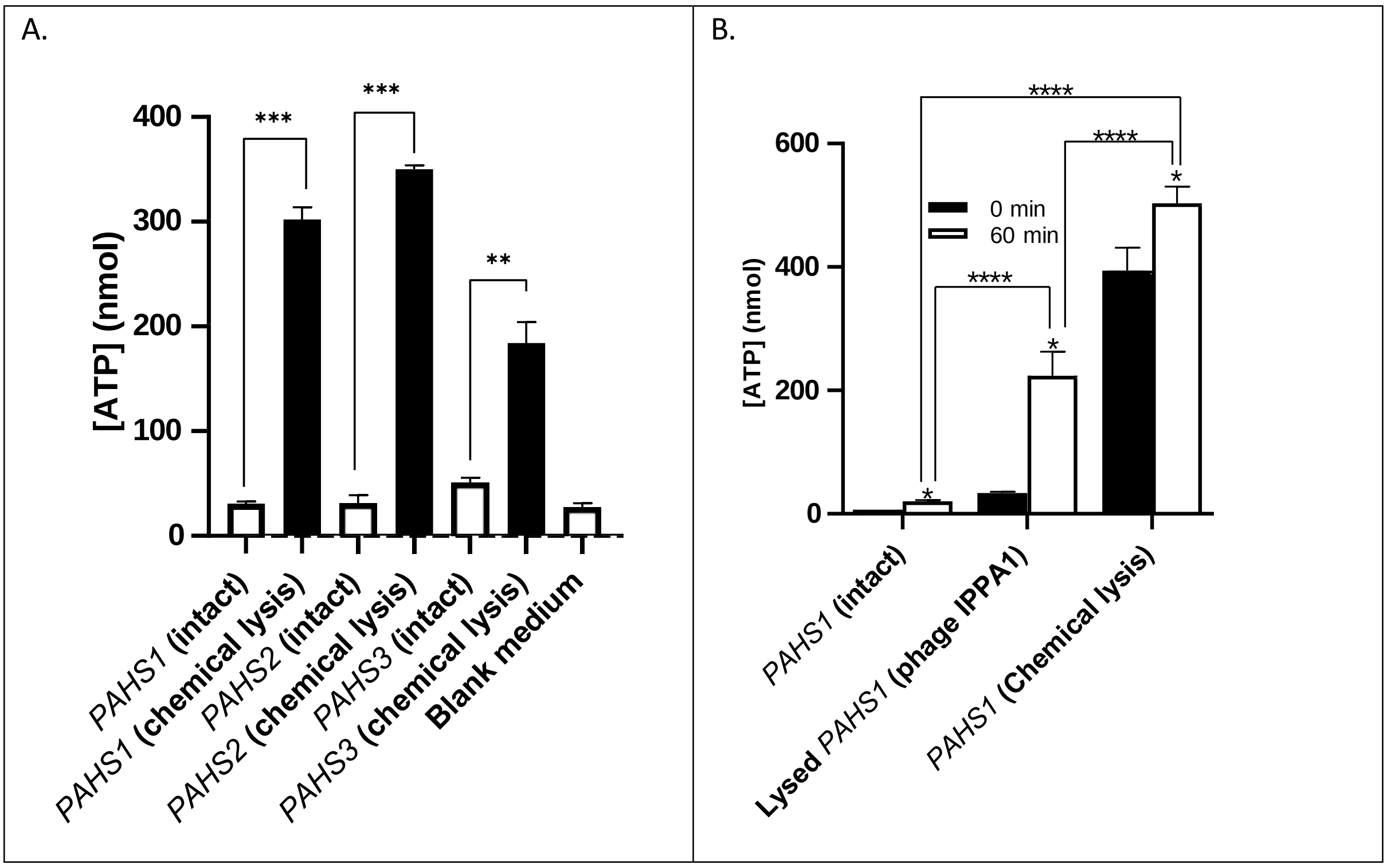

Figure S3: Mean with Standard Deviations for measured ATP concentrations for (**A)** three reference bacterial strains in liquid broth (LB), either intact (white bars) or chemically lysed (black bars), LB alone was used as control. **B.** ATP concentration in the medium after incubation of the *PAHS1* reference strains alone (intact bacteria); with the IPPA1 phage [10^8 PFU/mL]; and after chemical lysis. **: p-value <0.01; ***: p<0.001; ****p<0.0001; ns: non-significant.

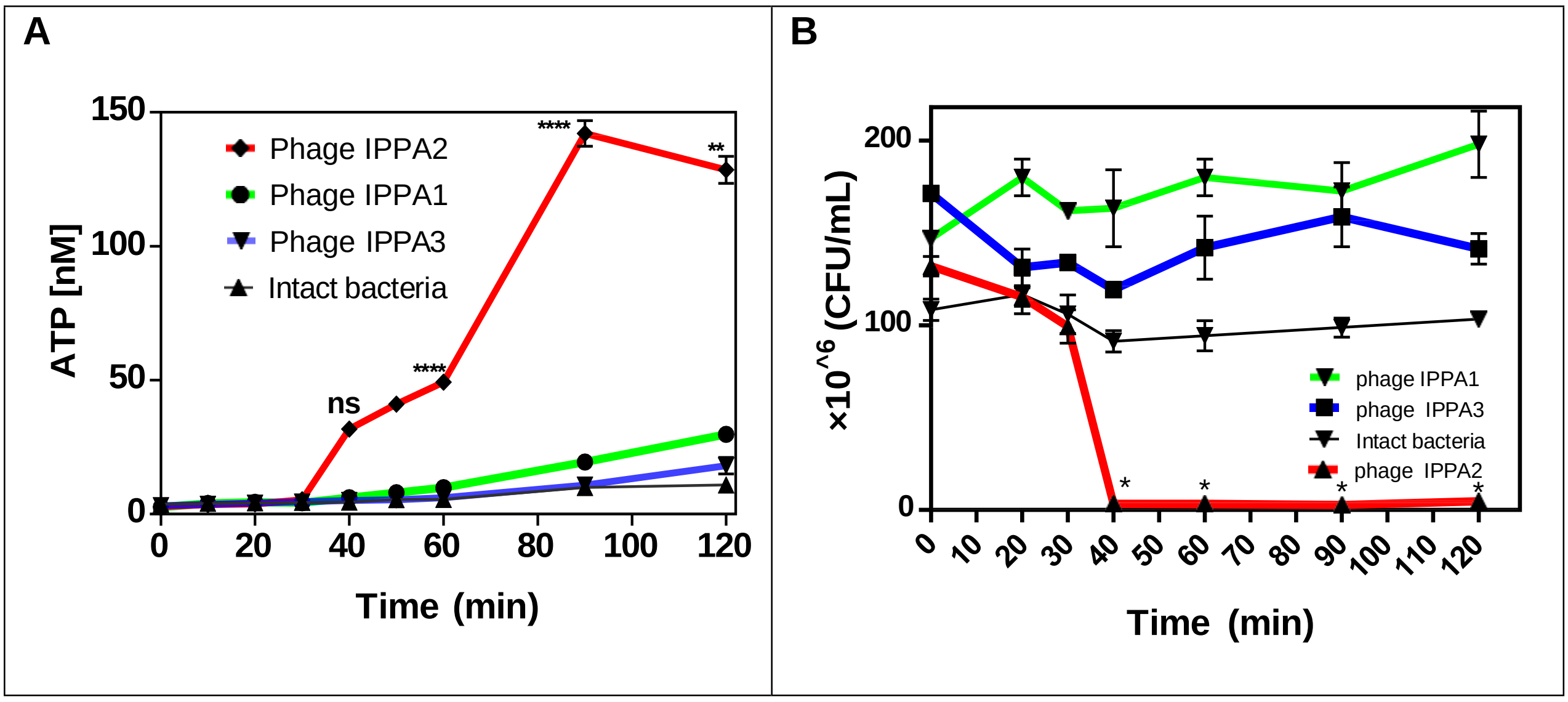

Figure S4: Mean with standard deviation for triplicates of **A.** [ATP] and **B.** bacterial colony count (CFU) for clinical strain 865 after 2 hours of incubation alone (black curve, used as negative control), in the presence of active phage IPPA2 (red curve) and inactive phages IPPA1 and IPPA3 (green and blue curves, respectively). ****, p-value < 0.0001; **, p-value < 0.005 (one-way ANOVA followed by post-hoc Dunnett's multiple comparison test); *, p-value < 0.05 (Mann-Whitney test).

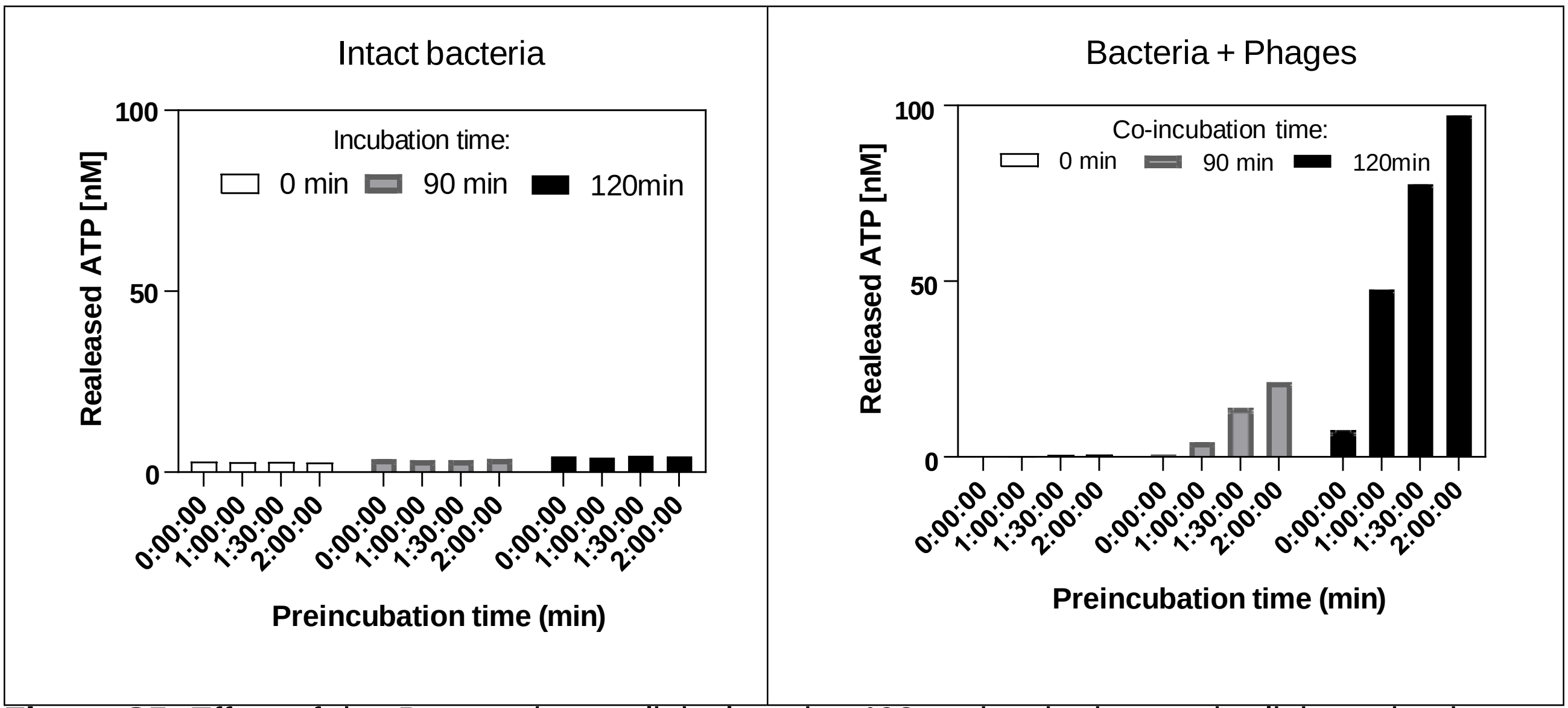

Figure S5: Effect of the *P. aeruginosa* clinical strain n°98 preincubation on the light emitted after (co-)incubation with a phage active against it (IPPA1). Bacteria were pre-incubated (x-axis) for 0, 60, 90 and 120 minutes alone for the indicated times prior to phage addition. After phage exposure, ATP release was measured following different co-incubation times: 0 min (white bars), 90 min (grey bars), and 120 min (black bars). The y-axis represents the amount of released ATP (nM).

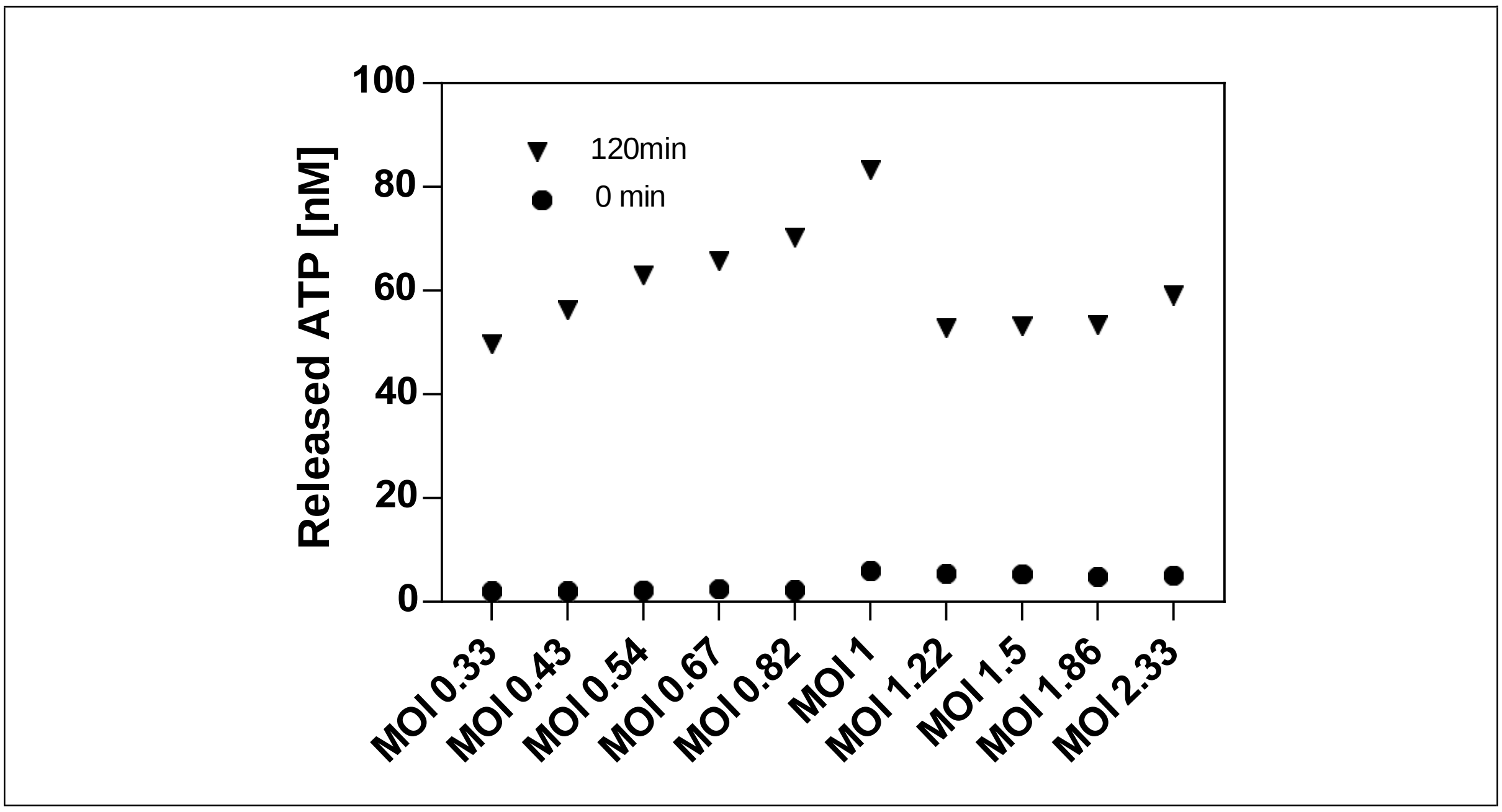

Figure S6: Released [ATP] after 120 min of co-incubation of various MOI (phage/bacteria ratio) of *P. aeruginosa* clinical strain 98 and a phage active against it (IPPA1).

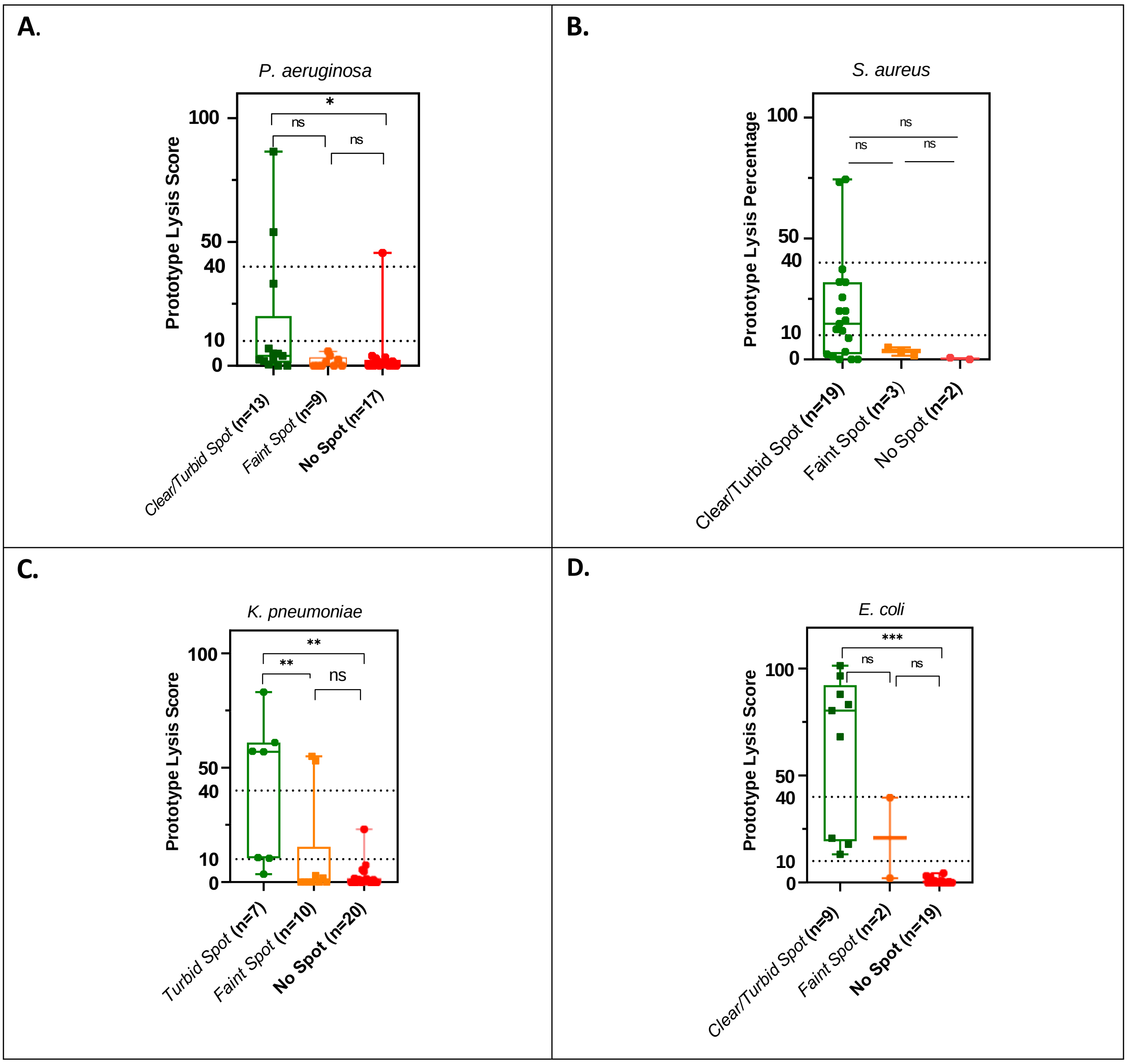

Figure S7: Lysis score obtained with the prototype as compared to ST results for DAO weak killers (EOP ≤ 0.005) for (**A**) *P. aeruginosa* (3 phages, 39 bacterial strain-phage pairs), **(B)** *S. aureus* (3 phages, 24 bacterial strain-phage pairs), **(C**) *K. pneumoniae* (2 phages, 37 bacterial strain-phage pairs), and (**D**) *E. coli* (2 phages, 30 bacterial strain-phage pairs)**.** Boxes indicate median, 25th and 75th percentiles, whiskers indicate minimum and maximum values; *: p-value <0.05; **: p<0.01; ***: p<0.001; ns: non-significant.

Table S1: Species specificity testing results for IPSA3 phage

Strains	a) RLU t60 (min) Bacteria with phages	b) RLU t60 (min) Intact bacteria	c) RLU t60 (min) Chemical lysis	Lysis score) =(a-b)/(c-b)
Ref strain S. aureus (SAHS2)	103 265	43 110	274 446	52
S. aureus ATCC29213	51 598	32 906	275 956	12,5
S. epidermidis 49341308	71 352	11 076	85 761	78,5
S. capitis ATCC 27840	13 5242	5 030	65 302	94
S. lugdunensis ATCC43809	39 435	2 081	37 814	62
S. haemolyticus ATCC 29970	2 885	2 773	26 948	0,5
S. anginosus LMG14502	120 429	154 732	165 142	-221,6
S. pyogenes ATCC19615	318 234	374 550	522 799	-45
S. agalactiae ATCC13813	365 385	430 300	525 991	-90
E. faecalis ATCC 29212	27 249	26 608	359 645	0
E. faecium IOWA1	32 615	33 976	291 103	-0,5
S. maltophilia ATCC13637	2 478	3 296	37 926	-2,5
S. marcescens 49329145	4 996	4 289	22019	7
K. oxytoca 2012B163	3 930	3 093	12971	10
K. pneumoniae ATCC700603	5 427	4 410	11 551	11
H. influenzae ATCC 49766	6 405	5 785	6 018	73
K. aerogenes ATCC13048	1 413	1 234	9 055	2
P. aeruginosa ATCC27853	2 052	1 955	5 717	2,5
P. mirabilis 30650446	1 224	1 229	1 984	-0,5
C. krusei IHEM65610	3 432	2 981	5 432	18
C. albicans ATCC24433	707	910	2 948	-8

Table S2. Results of PST performed on pellets of spiked urines

Strains	**Phages**	**Bacteriuria**	**CFU/ml**	**Prototype Lysis score**	**DAO**
PAHS1	IPPA1	++	4.5 x 10^7	104	0.3
		+++	9 x 10^7	93	0.3
SAHS1	IPSA3	++	1.3 x 10^8	71	1
		+++	1.9 x 10^8	72	1
K. pneumoniae 2706	IPKP1	++	3 x 10^7	47	2
		+++	8.8 x 10^7	66	2.6
E. coli 2060	IPEC1	++	3.5 x 10^7	98	0.7
		+++	8.6 x 10^7	90	1
